# Adaptive Tensor-Based Principal Component Analysis for Low-Dose CT Image Denoising

**DOI:** 10.1371/journal.pone.0126914

**Published:** 2015-05-18

**Authors:** Danni Ai, Jian Yang, Jingfan Fan, Weijian Cong, Yongtian Wang

**Affiliations:** Beijing Engineering Research Center of Mixed Reality and Advanced Display, School of Optics and Electronics, Beijing Institute of Technology, Beijing, 100081, China; Institute of Automation, Chinese Academy of Sciences, CHINA

## Abstract

Computed tomography (CT) has a revolutionized diagnostic radiology but involves large radiation doses that directly impact image quality. In this paper, we propose adaptive tensor-based principal component analysis (AT-PCA) algorithm for low-dose CT image denoising. Pixels in the image are presented by their nearby neighbors, and are modeled as a patch. Adaptive searching windows are calculated to find similar patches as training groups for further processing. Tensor-based PCA is used to obtain transformation matrices, and coefficients are sequentially shrunk by the linear minimum mean square error. Reconstructed patches are obtained, and a denoised image is finally achieved by aggregating all of these patches. The experimental results of the standard test image show that the best results are obtained with two denoising rounds according to six quantitative measures. For the experiment on the clinical images, the proposed AT-PCA method can suppress the noise, enhance the edge, and improve the image quality more effectively than NLM and KSVD denoising methods.

## Introduction

Since the inception of X-ray computed tomography (CT) in the 1790s, it has revolutionized diagnostic radiology and increased rapidly in usage [1]. CT utilizes computer-processed X-rays to produce tomographic images of specific areas of the scanned object. A 3D image of the interior of an object is generated from a large series of 2D radiographic images taken around a single axis of rotation. Thus, CT involves larger radiation doses than conventional X-ray imaging procedures. Moreover, X-rays indirectly or directly ionize DNA to cause strand breaks that are less easily repaired. Misrepair can occasionally induce point mutations, chromosomal translocations, and gene fusions, all of which are linked to cancer development [1]. Therefore, the use of diagnostic X-rays involves a low risk of developing cancer. Low individual risks applied to an increasingly large population may be a public health issue.

CT-related risks can be reduced through two ways. The first is by substituting CT with magnetic resonance imaging (MRI) or ultrasound. MRI has better descriptive powers than CT, but it cannot be used if metal is implanted in the body of the patient. It also produces low detailed images in bony structure examinations. MRI is more expensive than CT and ultrasound, and is available in specialized units. Ultrasound is cheaper than the other two methods, but detailed images are not obtained in ultrasound. The second is by reducing the radiation doses in individual patients and is the most effective way. However, the radiation dose directly influences image quality because of quantum statistics. A close relationship exists between pixel noise σ and radiation dose d [2], which is expressed as follows:

$$\sigma \propto \frac{1}{\sqrt{d}}$$ (1)

Ignoring the electronic noise caused by electronic devices is reasonable if up-to-date CT scanners are used. Thus, quantum statistics is the dominant source of noise. Eq 1 demonstrates that reducing the radiation dose increases pixel noise and degrades image quality. Providing an adequate image for clinical diagnosis with the minimum radiation dose is a major challenge in the CT field. The approaches to noise suppression can be classified into three categories: pre-processing, reconstruction, and post-processing. Pre-processing approaches decrease the directed noise and streak artifacts in the projected raw data [3]. However, filters cannot remove noise, or the noise reduction is accompanied by loss of image resolution [4]. Meanwhile, raw data are generally unavailable in a clinical environment. Reconstruction approaches optimize statistical objective functions by iterative numerical techniques [5]. In general, obtaining an appropriate image is time consuming. The aforementioned two approaches are beyond the scope of our research. This study focuses on post-processing approaches that suppress noise in reconstructed images.

Compared with standard-dose CT images (SDCT), low-dose CT images (LDCT) contain significantly more noise as described in Eq 1. A large amount of noise deteriorates the contrast and reduces the visibility of small objects. Both the noise and artifacts in LDCT cannot be modeled into one general distribution. It complicates the discrimination between noise and anatomy. Many denoising methods have been developed to improve the quality of images and control noise. These methods include bilateral filtering [6], total variation denoising [7], nonlocal means denoising [8] and K-SVD denoising [9]. In abdominal LDCT images, pixels with similar surrounding patches are likely in the same tissues; thus, [10] proposed a method to process pixel intensities by adaptively calculating the weighted intensity averaging of the pixels with similar surrounding structures throughout a large-scale neighborhood. In [11], window-based multi-wavelet transformation and thresholding are used to remove the additive white Gaussian noise in CT images. By estimating the local noise level, the adaptive nonlocal means filtering method proposed in [12] extends the original non-local means NLM and considers variation within and across slices of CT images.

Principal component analysis (PCA) is a linear subspace learning method that has been widely applied to reduce dimensionality by searching for the maximum variance directions. Recent studies have found that PCA can achieve image denoising excellently [13]. However, conventional PCA was originally proposed to process 1D vectors, which require all input data to first be unfolded into a vector to fit the nature of the PCA. This property may destroy the structure of the input data, such as the patches within an image that are always extracted to remove noise. This property may also cause overfitting problems because the dimension of the vectorized data may be larger than that of the sample number.

The present paper proposes adaptive tensor-based PCA (AT-PCA) to over come the limitation of PCA and remove the noise in the image. In this proposed method, “adaptive” has two meanings: (1) adaptively determining the size of the searching window to find similar patches with the objective patch and avoiding the grouping of completely different patches in the same stack; and (2) adaptively calculating the basis of PCA in different image locations to preserve the image edge structures after noise removal.

**Fig 1:**
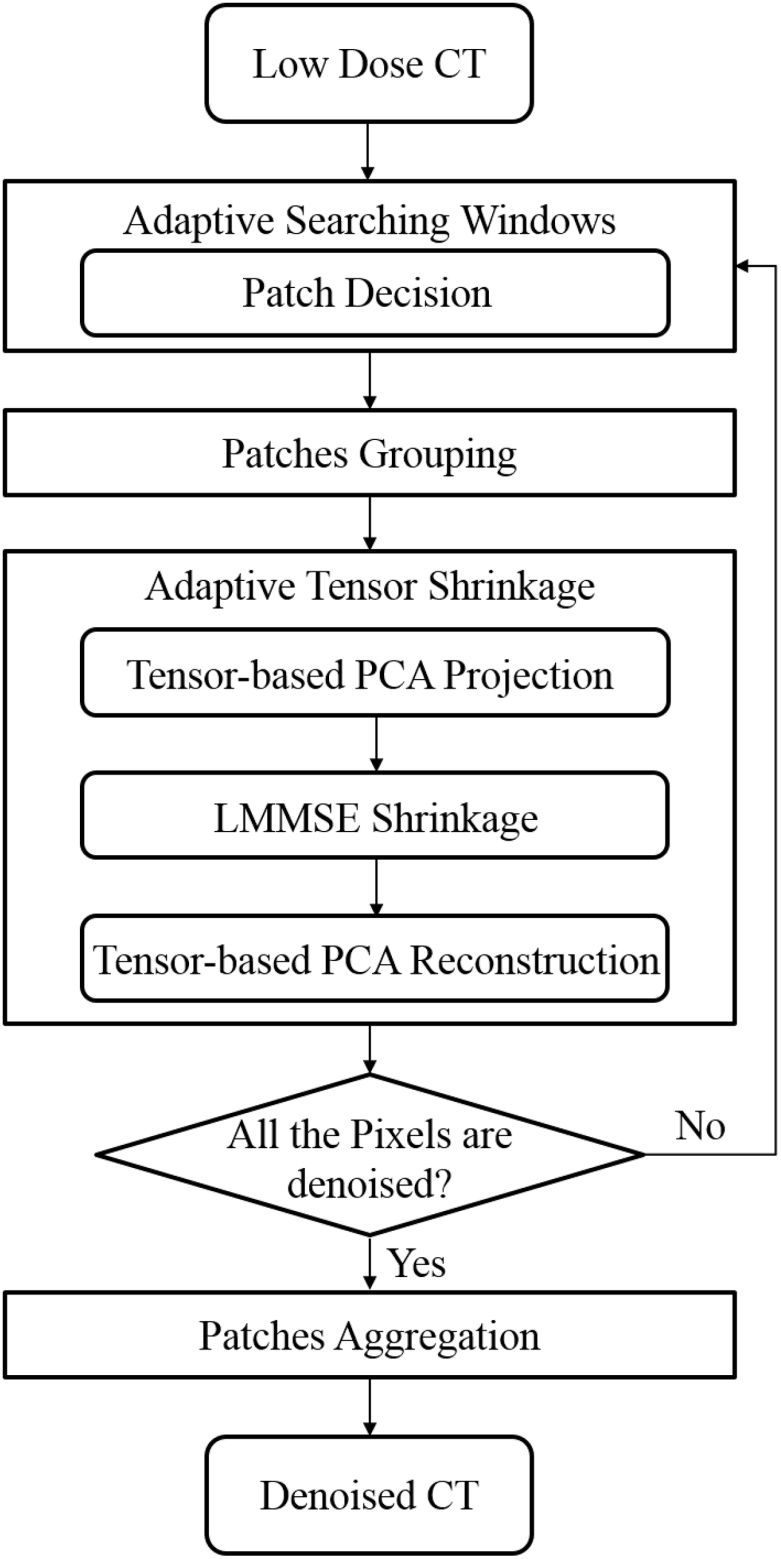
Flowchart of AT-PCA for low-dose CT denoising.

As shown in Fig 1, the proposed AT-PCA algorithm has two stages. The steps within each stage are similar, except that the noise level is updated after the first stage is finished because the initial estimation from the first stage is further refined by the second stage. Each stage consists of the following five steps: (1) similar patches are grouped within an adaptive searching window; (2) adaptive tensor-based PCA bases are calculated in terms of each similar patch group; (3) the linear minimum mean square error (LMMSE) is used to obtain the clean coefficients from local principal components; (4) shrinkage clean coefficients are employed for patch reconstruction; and (5) all reconstructed patches are aggregated, and the denoised image is finally obtained.

## Related Work

Principal component analysis is a fundamental linear subspace learning technique that uses an orthogonal transformation to convert a set of observations into a set of linearly uncorrelated variables. PCA mainly aims to reduce the dimension of the observations using uncorrelated variables and retain information that characterizes the variation in the observations as much as possible.

If we assume that **X** = [**x**_1_,**x**_2_,…,**x**_*N*_] denotes *N* training sample sets, we obtain the mean vector with$\bar{x}=\sum_{i=1}^{N}x_{i}/N$. To ensure that all samples are centered, we have${\tilde{x}}_{i}=x_{i}-\bar{x}$. Then, a set of orthonormal basis vectors $\tilde{U}=\left[u_{1},u_{2},\ldots ,u_{M}\right]$spanning an *m*-dimensional subspace is searched. The eigenvectors **u**_*m*_,(1≤*m*≤*M*) of the covariance matrix$C_{\tilde{x}}=E\left\{\tilde{x}{\tilde{x}}^{T}\right\}$ are the orthonormal basis vectors required by PCA. To complete the dimension reduction of data, eigenvectors **U** = [**u**_1_,**u**_2_,…,**u**_*k*_],(1≤*k<M*) that correspond to the largest *m* eigenvalues *λ*_1_≥*λ*_2_≥… *λ*_*m*_ are retained to minimize the mean-square error between ${\tilde{x}}_{i}$ and its reconstruction${\hat{x}}_{i}=\bar{x}+Uy_{i}$. In this equation, **y**_*i*_ is the *m*-dimensional uncorrelated variable of the original centered sample ${\tilde{x}}_{i}$ and is called principal component. Thus, the original sample is represented by a low-dimensional vector by projecting it into the PCA subspace. This representation can be expressed as follows:

$$y_{i}=U_{}^{T}\left({\hat{x}}_{i}^{}\text{−}\bar{x}\right)$$ (2)

As a linear subspace learning method, PCA requires the input data unfolded as vectors and may cause computation problems and destroy the structure of the input data. Furthermore, the number of the largest eigenvalues *m* is usually decided by the experience or Q-based method. It is not specific or adaptive to different denoising situations.

## Methodology

The proposed AT-PCA for CT image denoising contains four parts: adaptive searching windows design, similar patch grouping, adaptive tensor shrinkage, and patches aggregation. A reference patch centered on a pixel is first decided. Searching windows are adaptively designed to search similar patches as the reference patch and exclude patches with very different structures. In each searching window, tensor-based PCA transformation matrices are calculated by using the grouped similar patches as a training tensor. All the patches are projected into the tensor-based PCA subspace. In this subspace, LMMSE is used to shrink transformed components. Then, the shrinkage components of patches are reconstructed into the image space, and high-frequency noise is removed. After all patches sounding on each pixel is denoised, we aggregate the processed patches and obtain the denoised CT image. The flowchart of AT-PCA for low-dose CT denoising is shown in Fig 1.

### Adaptive Searching Window

**Fig 2:**
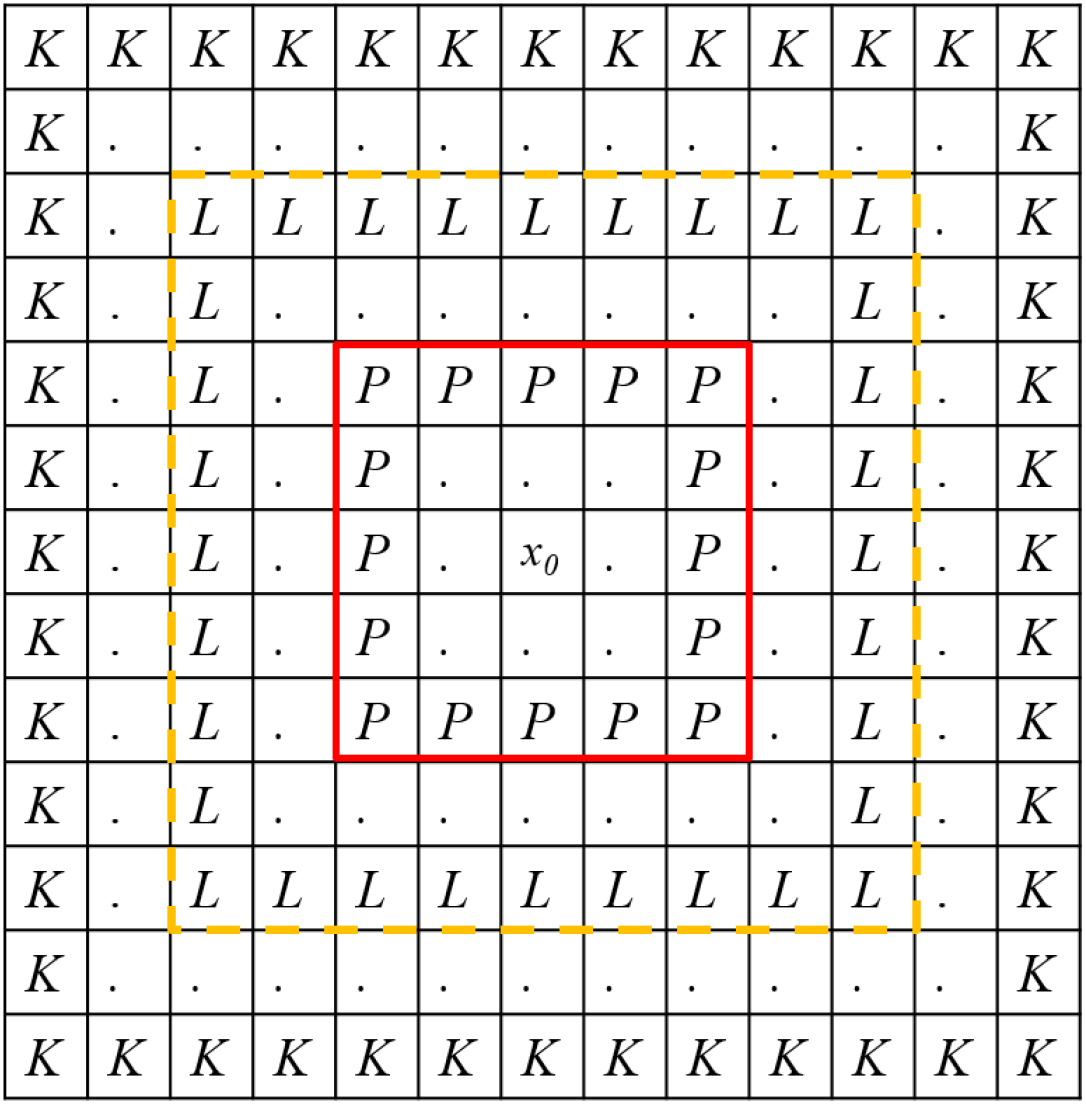
Diagrammatic sketch for hunting the adaptive searching window.

Patch-based image denoising has been widely used in recent research. Performing noise reduction on the patch (considering neighboring pixels) instead of the single pixel can preserve edge, which constitutes important semantic information of an image. Patch size is empirically decided and investigated in the experimental results of the study. In general, pixel denoising estimates the variable of its noisy observations within similar patches that can be searched around the entire image. However, this procedure is time consuming. To reduce the calculation time, we can search similar patches within a *L*×*L* window centered on the specific patch. This procedure is also based on the fact that similar patches are located near each other. This image has a complex structure, so an adaptive searching window with the size *L*×*L* is required for different locations of the specific patch. In Fig 2, the underlying pixel *x*_0_ to be noised is presented by a *P*×*P* patch shown in the red patch, denoted as **X**_0_∈*ℝ* ^*P*×*P*^. Similar patches [**X**_1_,**X**_2_,…,**X**_*N*−1_] as **X**_0_ are searched in a reasonable range with a size of *L*×*L* rather than in the whole image with a size of *Row*×*Col*. *L* is significantly smaller than both *Row* and *Col*. To decide the reasonable range for a searching window **B**∈*ℝ* ^*L*×*L*^, two types of evaluation standards are investigated within a few larger ranges of *K*×*K* than *L*×*L*. The evaluation standards include the median absolute deviation (MAD) and the inter-quartile range absolute deviation (IQRAD). Comparable ranges for deciding the optical size of **B** are limited between the range *P*+1 and the range *K*. All comparable searching windows can be unfolded as vectors and denoted as **b**_*i*_ = [*b*_1_,*b*_2_,…,*b*_*i*×*i*_], where *i*∈(*P*+1,*K*]. MAD is a robust scale estimator, and is presented as follows:

$$\text{MAD}\left(b_{i}\right)=\text{median}\left|b_{i}-\text{median}\left(b_{i}\right)\right|$$ (3)

where median(**b**_*i*_) is simply the middle order statistic when *L*×*L* is odd but the average of the order statistics with ranks $\frac{L\times L}{2}$ and $\left(\frac{L\times L}{2}+1\right)$ when *L*×*L* is even. IQRAD is a robust statistic that measures the difference between the upper and lower quartiles, as shown in Eq 3

$$\text{IQR}\left(b_{i}\right)=b_{i}{}_{Q_{3}}-b_{i}{}_{Q_{1}}$$ (4)

where $b_{i}{}_{Q_{1}}$ is the first quartile and $b_{i}{}_{Q_{3}}$ is the third quartile of **b**_*i*_. The adaptive size of the searching window is determined by

$$\text{SerW}\left(i\right)\text{=}\underset{i}{\min}\left(\left|\text{median}\left(X_{0}\right)\text{-MAD}\left(b_{i}\right)\right|+\left|\text{median}\left(X_{0}\right)\text{-IQR}\left(b_{i}\right)\right|\right)$$ (5)

where median(**X**_0_) is the value of centered pixel *x*_0_ to be noised, and *L* = *i* is the adaptive size of the searching window.

### Patch Grouping

Both spatial location and the intensity describe an image pixel that needs to be implemented by denoising. Thus, the noise of a whole image can be further removed. The semantic information of an image is contained by the edge structures that cause the high-frequency coefficients similar as noise. In general, the local edge structure can be represented by a pixel *x*_0_ and its nearest neighbors centered on *x*_0_, which is denoted as a patch **X**_0_∈*ℝ* ^*P*×*P*^. Denoising is performed on the patch instead of the single pixel. In the searching window with the adaptive size *L*, except for the target patch, we can find (*L*−*P*+1)^2^−1 patches **X**_*i*_,*i* = 1,…,(*L*−*P*+1)^2^, in which a significant difference from the target patch **X**_0_ may be contained. Patch grouping is implemented to select the patches that are similar to the target patch **X**_0_.

Methods such as clustering and matching can achieve patches grouping. The most popular clustering method is K-means, which aims to partition observations into k clusters. This is a hard clustering and each observation belongs to exactly one cluster. For a soft clustering, such as Fuzzy c-means [14], observations can belong to more than one cluster. In the process of clustering, recursive procedures are required, which are rather time consuming.

In our study, block matching is employed to select and group similar patches because of its simplicity and effectiveness. Block matching aims to find observations similar to a given reference, which meets our purpose. The patches around the underlying pixels are the references and similar observations located at different spatial locations can be found within the adaptive searching windows. If the distance between an observation and the reference is smaller than a given threshold, this observation can be considered similar to the reference and subsequently grouped. Distance can measure similarity between observations. Therefore, a smaller distance suggests a higher similarity. To numerically measure similarity of two observations, various distance measures can be used, such as Minkowski distance and Mahalanobis distance [15]. In the Euclidean space *ℝ* ^*N*^, which is the space of image patches, the distance between observations and the reference is usually given by the Euclidean distance (2-norm distance). The distance between the reference patch and others is presented as follows:

$$dist=\sqrt{\frac{1}{P\times P}\sum_{p=1}^{P\times P}{\left(X_{0}(p)-X_{i}(p)\right)}^{2}}$$ (6)

where *P*×*P* is the number of dimensions, and **X**_0_(*p*) and **X**_*i*_(*p*) are the *p* ^th^ data objects of **X**_0_ and **X**_*i*_, respectively. We select (*N*-1) similar patches [**X**_1_,,…,**X**_*N*−1_] with the shortest distances from the reference patch **X**_0_ and group all of them as a three-order tensor *X*∈*ℝ* ^*P*×*P×N*^, in which the patch size is exhibited in modes-1 and -2, and the training sample is displayed in mode-3.

### Adaptive Tensor Shrinkage

Based on the reasonable number of training patches as similar as the reference patch **X**_0_, tensor-based PCA can be used to remove the noise from **X**_0_. Patch grouping eliminates the large difference among training patches that may lead to the inaccurate estimation of the tensor-based PCA transformation matrix and further to noise residual. Unlike PCA which has to unfold the patch into a vector, tensor-based PCA directly processes the three-order tensor to obtain the transformation matrix for each mode. To this end, the elements of a three-order tensor must be stacked in a matrix to fit the algorithm of traditional PCA. In accordance with the definition of the matrix representation of a three-order tensor$X\in R^{I_{1}\times I_{2}\times I_{3}}$, the matrix unfolding $X_{\left(n\right)}\in R^{I_{n}\times (I_{n-1}I_{n+1})}$ on mode *n* contains the element $x_{i_{1}i_{2}i_{3}}$ at the position with row number *i*_*n*_ and column number equal to (*i*_*n+1*_−1)*I*_*n−1*_+ *i*_*n−1*_. The definitions of matrix unfolding involve the tensor dimensions *I*_1_,*I*_2_ and *I*_3_ in a cyclic way. In our case, **X**_(1)_∈*ℝ* ^*P*×(*PN)*^ and **X**_(2)_∈*ℝ* ^*P*×(*PN)*^, (*I*_1_ = *P*, *I*_2_ = *P*, *I*_3_ = *N*) are used. Thus, it overcomes the limitation that similar patches for training data are insufficient.

In accordance with three-order singular value decomposition, *χ* can be decomposed as the product

$$X=Y\times_{1}U^{\left(1\right)}\times_{2}U^{\left(2\right)}\times_{3}U^{\left(3\right)}$$ (7)

where **U** ^(1)^∈*ℝ* ^*P*×*P*^, **U** ^(2)^∈*ℝ* ^*P*×*P*^, and **U** ^(3)^∈*ℝ* ^*N*×*N*^ are unitary matrices, and *Υ*∈*ℝ* ^*P*×*P*×*N*^ is the core tensor. Thus, applied to a tensor *χ*, the orthogonal transformations of each mode can be found such that $Y=X\times_{1}U^{{\left(1\right)}^{T}}\times_{2}U^{{\left(2\right)}^{T}}\times_{3}U^{{\left(3\right)}^{T}}$is all-orthogonal and ordered [16].

In the tensor-based PCA transformed domain of *χ*, i.e. *Υ*, the energy of noiseless useful data is mostly concentrated on the several most significant components, whereas the energy of noise has a uniform distribution. To suppress the noise for different target patches with an adaptive shrinkage technique instead of a uniform dimension reduction, we use the linear minimum mean square-error estimation (LMMSE) technique in tensor-based PCA transformed domain. Let $y_{\left(n\right)}^{i},n=1,2$ be the *i* ^th^ column of **Y**_(*n*)_, which is the matrix unfolding in mold-*n* of *Υ*. The LMMSE of $y_{\left(n\right)}^{i}$ is obtained as follows:

$$\begin{array}{c} {\tilde{y}}_{i(n)}=w_{n}\cdot y_{\left(n\right)}^{i}\\ =\left[w_{n}(1)w_{n}(2)\cdots w_{n}(P)\right]\cdot \left[y_{\left(n\right)}^{i}(1)y_{\left(n\right)}^{i}(2)\cdots y_{\left(n\right)}^{i}(P)\right] \end{array}$$ (8)

In Eq 3, the shrinkage coefficient is calculated as follows:

$$w_{n}(p)=\frac{E\left[{\left(x_{\left(n\right)}^{i}(p)\right)}^{2}\right]}{E\left[{\left(x_{\left(n\right)}^{i}(p)\right)}^{2}\right]+E\left[{\left(v_{\left(n\right)}^{i}(p)\right)}^{2}\right]}$$ (9)

where $E\left[{\left(x_{\left(n\right)}^{i}(p)\right)}^{2}\right]$ and $E\left[{\left(v_{\left(n\right)}^{i}(p)\right)}^{2}\right]$ are the signal and noise variances, respectively, and $x_{\left(n\right)}^{i}$ is the component of the *i* ^th^ column of **X**_(*n*)_, which is the matrix unfolding in mold-*n* of *χ*. Using the roust median estimator of the highest sub-band of a Daubechies two wavelet transform, we can estimate the noise variance as follows [17]:

$$E\left[{\left(v_{\left(n\right)}^{i}(p)\right)}^{2}\right]={\left(\frac{\text{Median}(HH)}{0.6745}\right)}^{2}$$ (10)

where *HH* is the high-high band wavelet coefficients. The signal variance is estimated using the maximum likelihood estimator as follows:

$$E\left[{\left(x_{\left(n\right)}^{i}(p)\right)}^{2}\right]=\max\left[0,\frac{1}{M}\sum_{i=1}^{M}\left({\left(y_{\left(n\right)}^{i}(p)\right)}^{2}-E\left[{\left(v_{\left(n\right)}^{i}(p)\right)}^{2}\right]\right)\right]$$ (11)

where *M* is the column number of **Y**_(*n*)_. In flat zones, $\left({\left(y_{\left(n\right)}^{i}(p)\right)}^{2}-E\left[{\left(v_{\left(n\right)}^{i}(p)\right)}^{2}\right]\right)$is often less than zero, so that *w*_*n*_(*p*) becomes 0 and the noise in $y_{\left(n\right)}^{i}$ is removed as${\tilde{y}}_{i(n)}$.

### Tensor Reconstruction

Let *W* ^(n)^∈*ℝ* ^*P*×*P*×*N*^ have **w**_*n*_ in each column. The shrunk $\tilde{Y}$ is expressed as follows:

$$\tilde{Y}=Y\cdot W^{\left(1\right)}\cdot W^{\left(2\right)}$$ (12)

where (·) denotes the inner product of tensors. The denoised result of *χ* can be obtained by transforming $\tilde{Y}$ in the tensor-based domain back to the time domain, which is shown as follows:

$$\tilde{X}=\tilde{Y}\times_{1}U^{\left(1\right)}\times_{2}U^{\left(2\right)}\times_{3}U^{\left(3\right)}$$ (13)

The denoised estimation of the reference patch **X**_0_, denoted as${\tilde{X}}_{0}$, can be obtained from$\tilde{X}$. The final denoised result of the underlying central pixel *x*_0_, denoted as${\tilde{x}}_{0}$, can be extracted from${\tilde{X}}_{0}$. For a whole image$I\in R^{M_{1}\times M_{2}}$, *M*_1_×*M*_2_ pixels should be denoised by applying the aforementioned procedure, which leads to the full denoised image of **I**, which is$\bar{I}\in R^{M_{1}\times M_{2}}$. Each pixel in an image has many denoised values because reference patches representing the underlying central pixels are overlapping. An accumulation is performed by averaging all of the overlapping denoised patches for a final value of denoised pixels, such that we obtain

$$\bar{I}=\frac{\sum_{i\in [1,M_{1}\times M_{2}]}\sum_{x_{R}}{\tilde{X}}_{i}^{x_{R}}}{\sum_{i\in [1,M_{1}\times M_{2}]}\sum_{x_{R}}E_{i}^{x_{R}}}$$ (14)

where **E**_*i*_ has the same size as ${\tilde{X}}_{i}$ and all elements of **E**_*i*_ are one, and *x*_*R*_ is the coordinate iterated in patches. This process generates a denoised image.

### Quantitative Measures

The following six metrics as quantitative performance are used to assess the quality of the images: standard deviation (STD) [18], mean-square error (MSE) [19], signal-to-noise ratio (SNR) [20], peak signal-to-noise ratio (PSNR) [21], structural similarity (SSIM) [22], and parameter *β* [23]. The calculation functions are illustrated as follows:

1. STD:
  STD=(∑x,y∈ROIs(I¯¯(x,y)−mean(I¯¯(x,y)))2|ROIs|−1)12 (15)
  where |ROIs|is the pixel number in the regions of interest (ROIs). For the standard test image, the ROI is the whole image.
2. MSE:
  $$\text{MSE}=\frac{1}{M_{1}N_{1}}\sum_{x=1}^{M_{1}}\sum_{y=1}^{N_{1}}{\left|\tilde{I}\left(x,y\right)-\bar{\bar{I}}\left(x,y\right)\right|}^{2}$$ (16)
  where $\tilde{I}\in R^{M_{1}\times M_{2}}$ is the pure image without any disturbing noise.
3. SNR:
  $$\text{SNR}=10\;\cdot \;\log_{10}\frac{\frac{1}{M_{1}N_{1}}\sum_{x=1}^{M_{1}}\sum_{y=1}^{N_{1}}{\left(\tilde{I}\left(x,y\right)\right)}^{2}}{\text{MSE}}$$ (17)
4. PSNR:
  $$\text{PSNR}=10\cdot \log_{10}\frac{N_{\max}}{\text{MSE}}$$ (18)
  where *N*_max_ is the maximum fluctuation in the input image.
5. SSIM:
  $$\text{SSIM}=\frac{\left(2\mu_{\tilde{I}\left(x,y\right)}\mu_{\bar{\bar{I}}\left(x,y\right)}+C_{1}\right)\left(2\sigma_{\tilde{I}\left(x,y\right)\bar{\bar{I}}\left(x,y\right)}+C_{2}\right)}{\left(\mu_{\tilde{I}\left(x,y\right)}^{2}+\mu_{\bar{\bar{I}}\left(x,y\right)}^{2}+C_{1}\right)\left(\sigma_{\tilde{I}\left(x,y\right)}^{2}+\sigma_{\bar{\bar{I}}\left(x,y\right)}^{2}+C_{2}\right)}$$ (19)
  where$\mu_{\tilde{I}\left(x,y\right)}$, $\mu_{\bar{\bar{I}}\left(x,y\right)}$, $\sigma_{\tilde{I}\left(x,y\right)}^{2}$, $\sigma_{\bar{\bar{I}}\left(x,y\right)}^{2}$, and $2\sigma_{\tilde{I}\left(x,y\right)\bar{\bar{I}}\left(x,y\right)}$ are the means of $\tilde{I}$ and$\bar{\bar{I}}$, the variances of $\tilde{I}$ and$\bar{\bar{I}}$, and the covariance of $\tilde{I}$ and$\bar{\bar{I}}$, respectively; *C*_1_ and *C*_2_ are small constants.
6. Parameter *β* given by [23] is indented to assess the ability of the denoising method to preserve sharp details of the images:
  $$\beta =\frac{\langle \left(\Delta \tilde{I}-\bar{\Delta }\tilde{I}\right),\left(\Delta \bar{\bar{I}}-\bar{\Delta }\bar{\bar{I}}\right)\rangle }{\sqrt{\langle \left(\Delta \tilde{I}-\bar{\Delta }\tilde{I}\right),\left(\Delta \tilde{I}-\bar{\Delta }\tilde{I}\right)\rangle \langle \left(\Delta \bar{\bar{I}}-\bar{\Delta }\bar{\bar{I}}\right),\left(\Delta \bar{\bar{I}}-\bar{\Delta }\bar{\bar{I}}\right)\rangle }}$$ (20)
  where $\Delta \tilde{I}$ and $\Delta \bar{\bar{I}}$ are the high-pass filtered images of $\tilde{I}$ and$\bar{\bar{I}}$; 〈**·**,**·**〉 is the standard inner-product. The closer to 1, the better is the ability of denoising to retain the image edges.

## Experimental Results

Two types of image data are utilized to investigate the effect of denoising: one standard test image and a group of clinical images. “House” image, which is widely used in the image processing literature, is used for quantification. It has a size of 256×256. All the clinical images used in this study are provided by the Laboratory of Image Science and Technology, Southeast University, and the study on these data was approved by the institutional ethical review board of the university [24–26]. The patients involved in our study (two females and one male aged 63, 38, and 53, respectively) provide written consent. All patients suffered from cancer as confirmed by biopsy examinations. All of the CT images were acquired on a 16-channel multi-detector row CT scanner (Somatom Sensation 16; Siemens, Forchheim, Germany). A reduced tube current of 50 mAs and a routine tube current of 260 mAs were used to acquire LDCT and SDCT images, which were exported as DICOM data with a size of 512×512.

Two-round denoising has recently become more popular than one-round processing. In our study, the times of the denoising round are investigated on the standard test image based on six metrics. The best time is set for the clinical images. Further processing is performed using a standard PC with MATLAB as the developing language.

### Standard Test Image Experiments

The denoising procedures described in Section 3.1–3.3 can remove most of noise. However, the signal variances directly calculated from the noise disturbed image may not be accurately evaluated because of the strong noise contained in the original image. This result consequently leads to the estimation bias of the shrinkage coefficient. Several visually unpleasant noise residuals remained. Therefore, a second round of the aforementioned denoising procedure is necessary to further enhance the denoising result obtained from the first round. The noise level in the second stage should be renewed because most noise has been eliminated in the first stage. In our study, the noise variance of the input image in the second stage is estimated as follows [27]:

$$E\left[{\left({\hat{v}}_{\left(n\right)}^{i}(p)\right)}^{2}\right]\text{=0}.36\times E\left[{\left(v_{\left(n\right)}^{i}(p)\right)}^{2}\right]$$ (21)

Then, all the procedures mentioned in Sections 3.1–3.3 are executed with the output of the first round and renewed noise level. In this section, four denoising rounds are investigated to obtain the best round for the final denoised image$\bar{\bar{I}}\in R^{M_{1}\times M_{2}}$.

**Fig 3:**
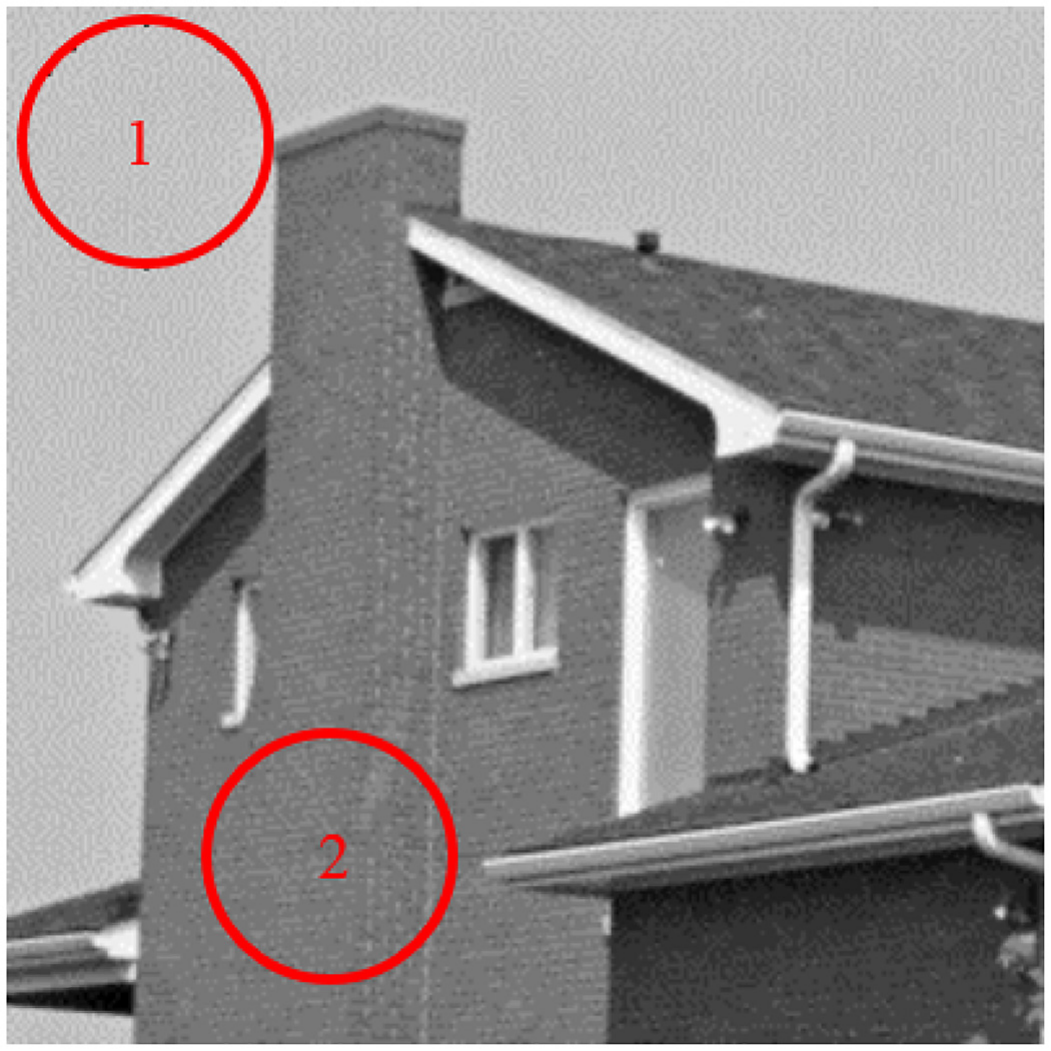
Illustration of regions of interest for STD calculation.

After adding the Gaussian white noise of *σ* = 40 to the original “House” image, we investigate six metrics for noise removal. Two regions of interest for STD calculation are marked in Fig 3. Table 1 indicates the STDs of different ROIs for the original image, the noisy image, and the denoised images in the four stages. The values of STD decrease as the number of rounds increases. A low STD indicates that the data points are very close to the mean, whereas a high STD indicates that the data points are spread over a large range of values. In Fig 3, region 1 is smooth, but region 2 contains texture information. Thus, the value of STD is lower in the region 1 than in region 2. In the first-round denoising stage, the values of STD have already been dramatically reduced. The remaining three rounds reduce the STD subtly.

**Table 1:** Standard deviations of different ROIs for the original image, noisy image, and denoised images in the four stages.

STD	Original Image	Noisy image	First stage	Second stage	Third stage	Fourth stage
1	1.8175	19.1115	3.0910	2.1191	1.6626	1.6863
2	6.9991	20.2324	4.0612	3.2579	2.8147	2.8324

**Fig 4:**
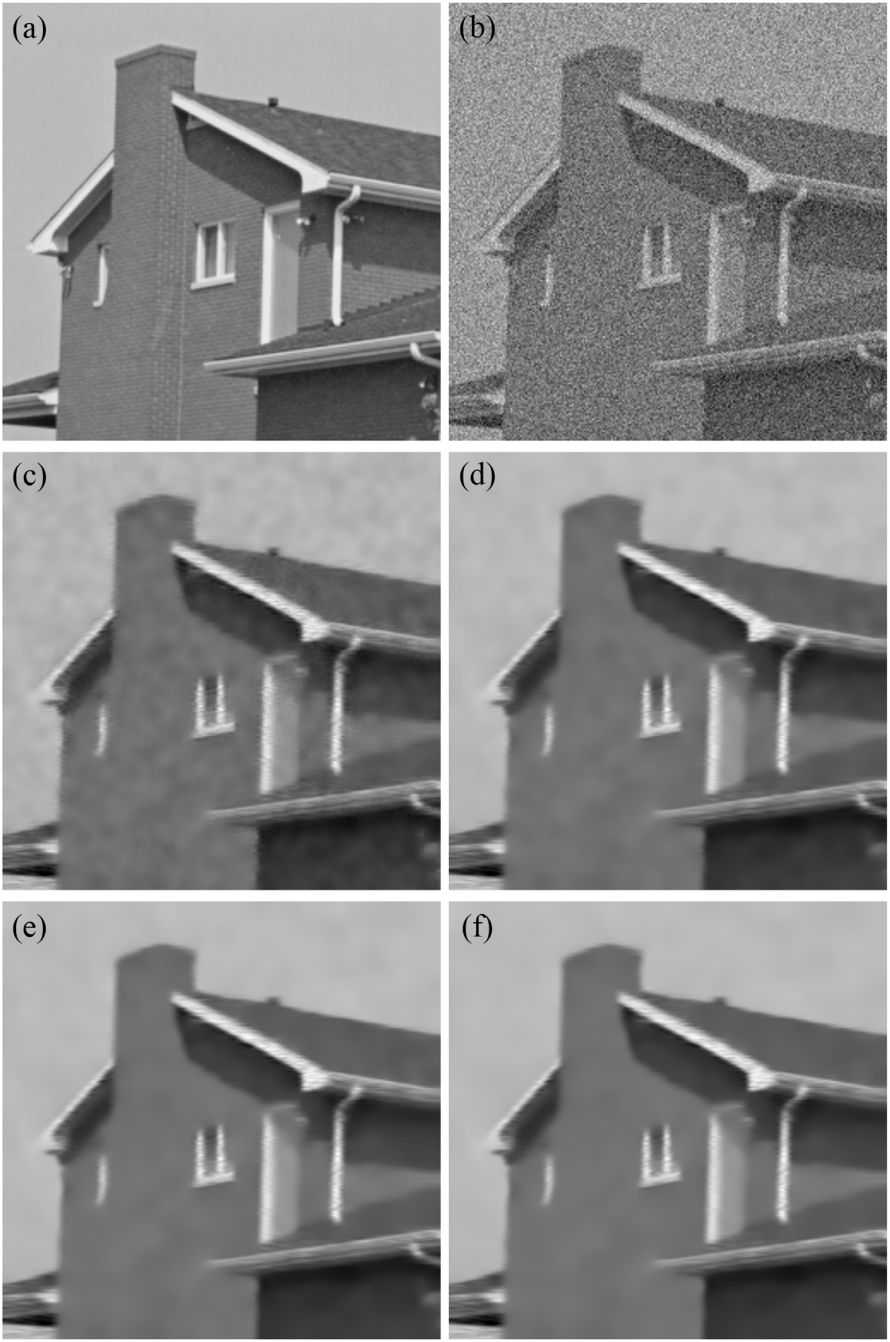
Denoised results of the “House” image at different stages. (a) Original House image; (b) Noisy image; (c) Denoised result in the first stage; (d) Denoised result in the second stage; (e) Denoised result in the third stage; (f) Denoised result in the fourth stage.

**Fig 5:**
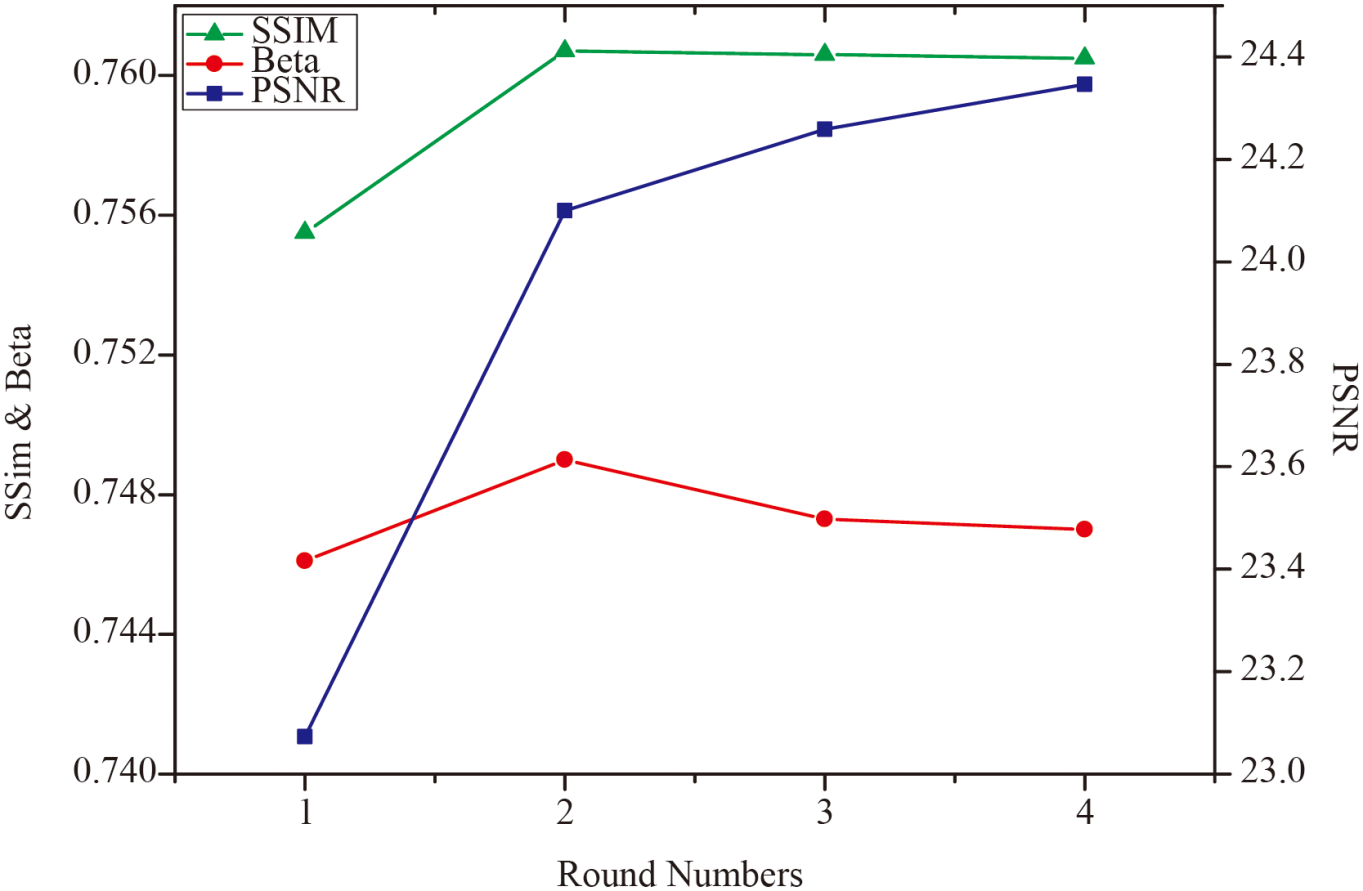
Variation trend of SSIM, Beta, and PSNR in different rounds.

Table 2 shows the MSE, SNR, PSNR, SSIM and *β* results of the noisy “House” image and denoised images in the four procedure stages. After the procedure in the first-stage MSE dramatically decreases, the SNR, PSNR, SSIM, and *β* obviously increase. Thus, most noise has been removed by using only one denoising round. These five metrics are further upgraded in the second round. Then the denoising ability and edge preservation are both enhanced. Fig 4b to 4d show the same performance, that is, the first round for Fig 4b obtains a more impressive denoising result of Fig 4c, and the second round for Fig 4b refines Fig 4c to 4d. Furthermore, the MSE decreases while the SNR and PSNR slightly increase for the third and fourth stages, respectively (Table 1). The denoising result is better in the third and fourth stages than in the first stage. However, the values of SSIM and *β* gradually reduce. SSIM measures the similarity between two images and considers image degradation as perceived change in structural information. *β* is a measure of edge preservation, which is a significant piece of information for denoising investigation. Decreases in both SSIM and *β* indicate that more rounds may not improve the effect of denoising. Fig 5 shows the variation trend of SSIM, *β*, and PSNR in different rounds. Thus, two rounds are accepted in denoising in further clinical studies.

**Table 2:** MSE, SNR, PSNR, SSIM, and *β* results of the noisy “House” image and denoised images in the four stages.

σ = 40	Noisy image	First stage	Second stage	Third stage	Fourth stage
MSE	1310	320.47	252.97	243.91	239.04
SNR	60.46	66.58	67.61	67.76	67.85
PSNR	16.96	23.07	24.10	24.26	24.35
SSim	0.2834	0.7555	0.7607	0.7606	0.7605
Beta	0.2647	0.7461	0.7490	0.7473	0.7470

### Clinical Experiments

#### Quantification of Patch Size

**Fig 6:**
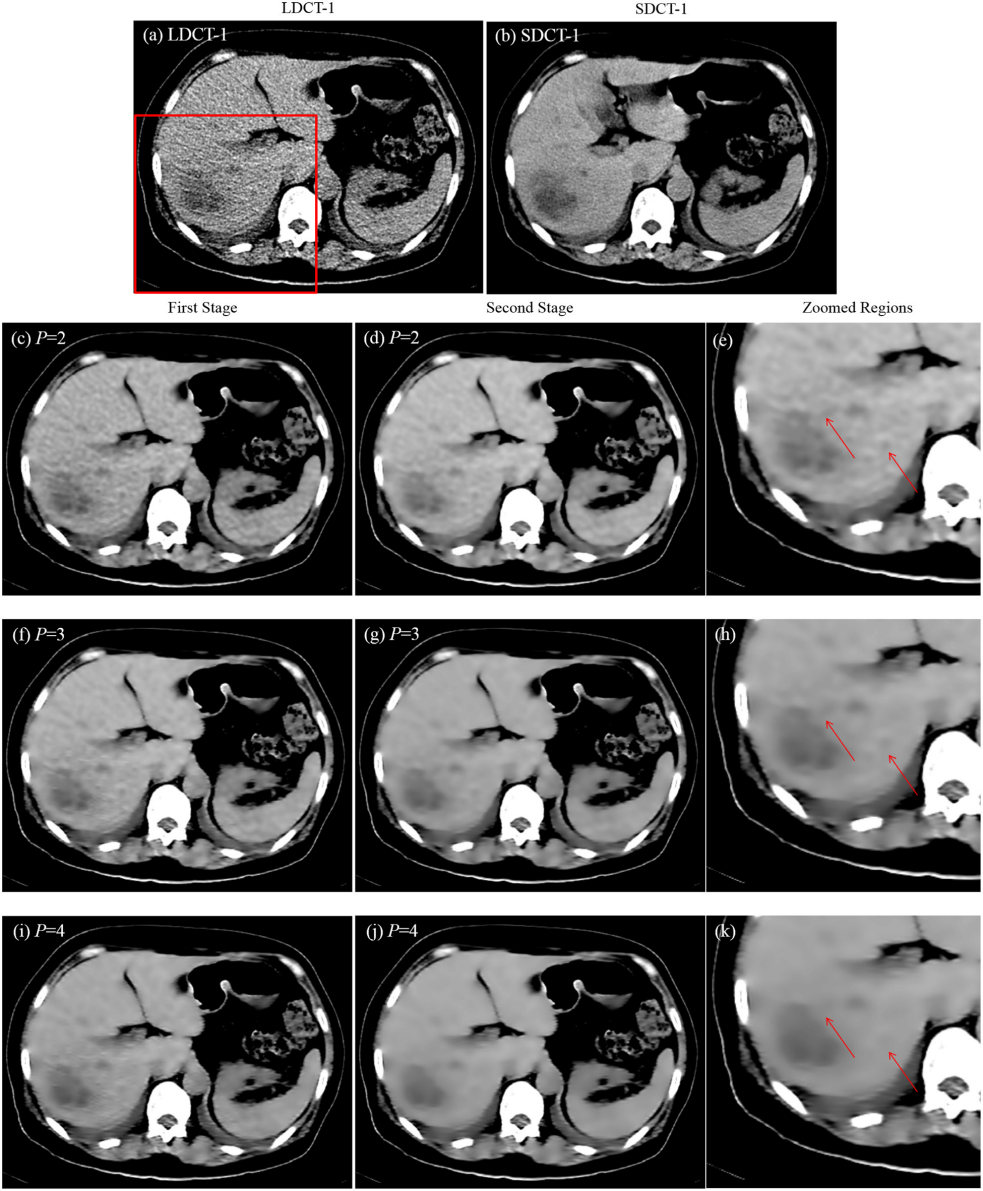
Denoising results with three parameters and two stages. (a) LDCT image; (b) SDCT image; (c), (f), and (i) are the LDCT images denoised with *P* = 2, *P* = 3, and *P* = 4 in the first stage, respectively; (d), (g), and (j) are the LDCT images denoised with *P* = 2, *P* = 3, and *P* = 4 in the second stage, respectively; (e), (h), and (k) are the zoomed regions specified in (a).

Most of the parameters in our algorithm are automatically set in accordance with the character of the specific image. However, one parameter is left for adjustment. This parameter is the size of patch *P* for presenting the underlying pixel to be denoised. We investigate three values of *P* (*P* = 2, *P* = 3, and *P* = 4) to compare the denoising results and select the best parameter that can remove visually unpleasant noise residual and retain tumor information. In Fig 6, the first row displays the LDCT and SDCT directly obtained from a CT scanner. Considering that LDCT and SDCT are produced by two scans that cause unavoidable displacements, we select the most similar visual SDCT slice as the LDCT slice, which is shown in Fig 6b. The second to the fourth rows indicate the results of the LDCT image obtained in the first stage on the left column (Fig 6c, 6f and 6i) and the corresponding results obtained in the second stage on the middle column (Fig 6d, 6g and 6j). After the refinement in the second stage, visually unpleasant noise residual is noticeably removed. Zoomed regions specified in Fig 6e, 6h and 6k are further shown in the third column. The tumors indicated by the arrows are more clearly presented in the second stage with *P* = 3. Thus, noise is suppressed and significant tumor information is retained within *P* = 3 in the second stage.

#### Quantitative Assessment

As described in Section 3, six metrics can be used to evaluate the quantitative performance of different denoising algorithms for images whose purely images without any disturbing noise can be obtained simultaneously. However, in the clinical case, no exact spatial correspondence exists between LDCT and SDCT in two different scans because of the unavoidable displacements caused by the breath or body movements of patients. The metrics based on Euclidean distance cannot be used for the quantitative evaluation of CT image quality. In this study, only the STD of the ROIs is used to compare the effect of denoising in LDCT, processed LDCT, and selected corresponding SDCT.

**Fig 7:**
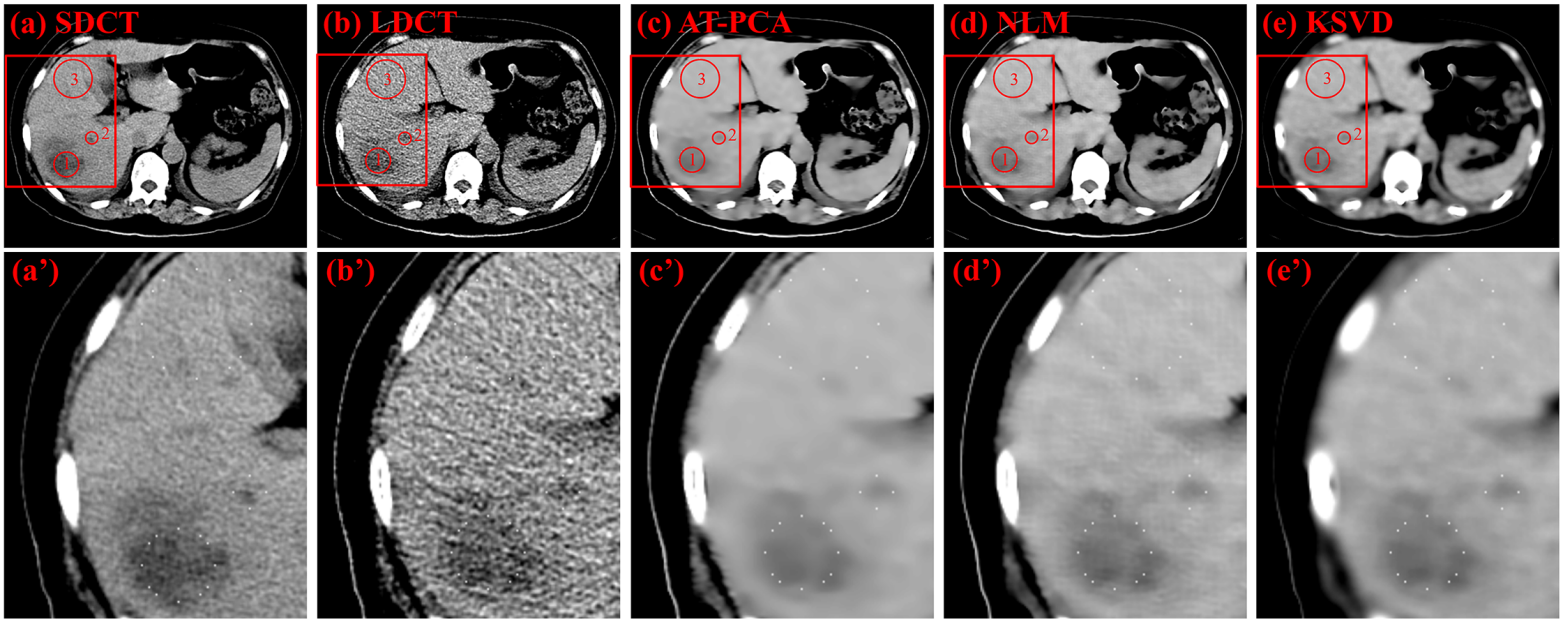
Denoising results for a 53-year-old female patient with liver tumor. The first column from (a) to (e) shows the SDCT image, the LDCT image, as well as the images denoised with AT-PCA, NLM and KSVD, respectively. Red circles and numbers denote positions of tumors (numbers 1 and 2) and the background (number 3). The second column from (a’) to (e’) shows the zoomed regions corresponding to (a) to (e).

**Fig 8:**
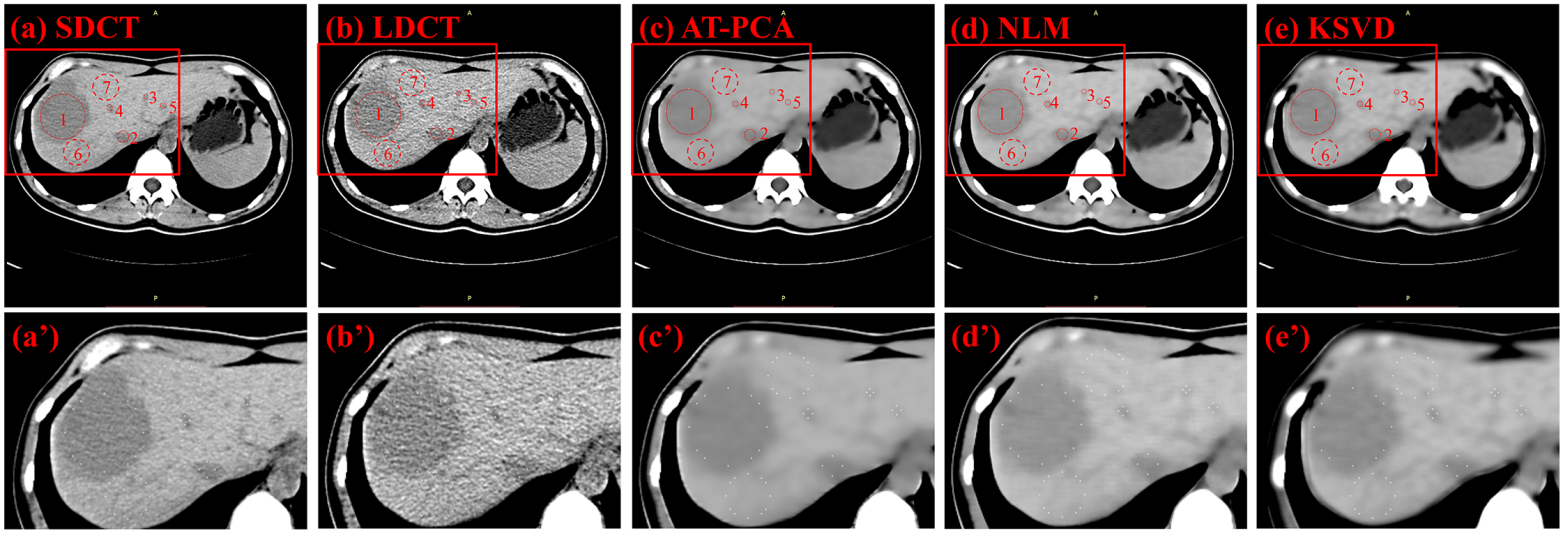
Denoising results for a 61-year-old female patient with liver tumor. The first column from (a) to (e) shows the SDCT image, LDCT image, and images denoised with AT-PCA, NLM and KSVD, respectively. Red circles and numbers denote positions of tumors (numbers 1, 2, 3, 4, 5) and the backgrounds (numbers 6 and 7). The second column from (a’) to (e’) shows the zoomed regions corresponding to (a) to (e).

Two types of CT images are used for denoising comparison: those is from the 53-year-old (Fig 7) and 61-year-old (Fig 8) female patients with liver tumors. Three ROIs are highlighted in Fig 7, with two of them (numbers 1 and 2) indicating the tumors and one (number 3) designating the background. The original SDCT and LDCT images are presented in Fig 7a and 7b. The processed images based on the algorithms of AT-PCA, NLM, and KSVD are shown in Fig 7c to 7e. In the second column, the zoomed regions corresponding to the Fig 7a to 7e are displayed and labeled Fig 7a’ to 7e’. Compared with the SDCT image, the LDCT image shows that the tumors are merged by intense noise. After the denoising algorithms are implemented, the noise can be effectively reduced and quantitatively assessed as Table 3. In Table 3, the highest STDs are obtained from the LDCT image either for the tumors or for the background, which is consistent with the fact that a low dose leads to high noise and degrades image quality. The proposed AT-PCA can obtain the lowest STDs for both tumors and background. It only removes the redundant noise; no additional artifact is introduced unlike in the NLM case (Fig 7d’), or the edge is enhanced unlike in the KSVD case (Fig 7e’). The same experimental results can be achieved in Fig 8 and Table 4. Seven ROIs, including five tumor regions and two background regions, are highlighted; the corresponding STDs are quantitatively calculated and presented in Table 4. Equally, the lowest STDs for both tumors and background are calculated by the proposed method. Since the NLM case introduces the artifacts and the KSVD case blurs the edge, all of the STDs for these cases are higher than those for the AT-PCA case.

**Table 3:** STDs of three ROIs for LDCT, SDCT, and processed LDCT images of a 53-year-old female patient with liver tumor.

ROIs	LDCT	SDCT	AT-PCA	NLM	K-SVD
1	10.65	5.82	2.61	3.21	3.08
2	12.43	6.47	2.97	3.87	3.57
3	9.91	4.41	1.04	1.55	1.60

**Table 4:** STDs of seven ROIs for LDCT, SDCT, and processed LDCT images of a 61-year-old female patient with liver tumor.

ROIs	LDCT	SDCT	ST-PCA	NLM	K-SVD
1	7.93	3.74	0.63	0.94	1.05
2	8.78	5.83	2.10	2.82	2.95
3	7.25	3.60	0.68	1.31	1.10
4	7.42	5.96	1.17	1.34	1.46
5	6.01	5.89	0.60	1.33	1.02
6	7.61	4.28	1.15	1.27	1.41
7	7.83	3.67	0.57	1.22	1.42

#### Visual Assessment

**Fig 9:**
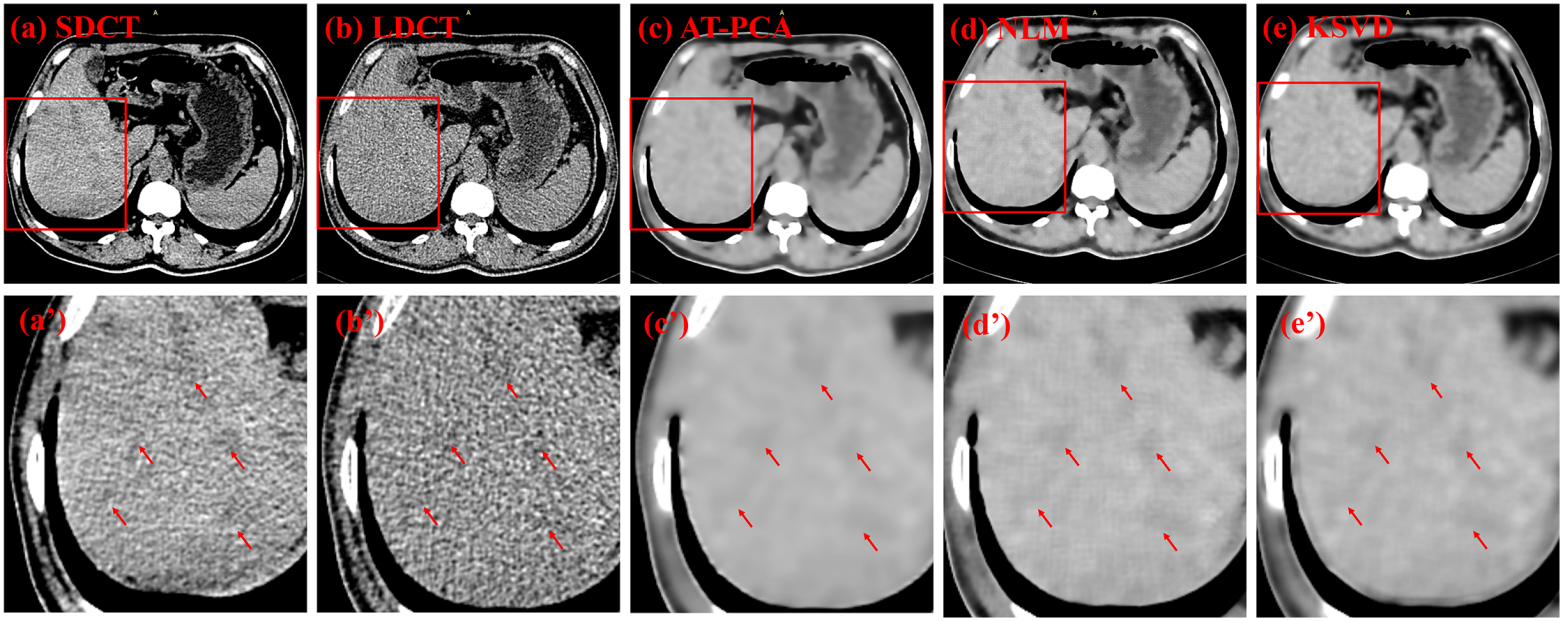
Denoising results for a 56-year-old male patient with multiple hepatic metastases (slice 1). The first row from (a) to (e) shows the SDCT image, the LDCT image, and the images denoised with AT-PCA, NLM, and KSVD, respectively. The second row from (a’) to (e’) shows the zoomed regions corresponding to (a) to (e).

**Fig 10:**
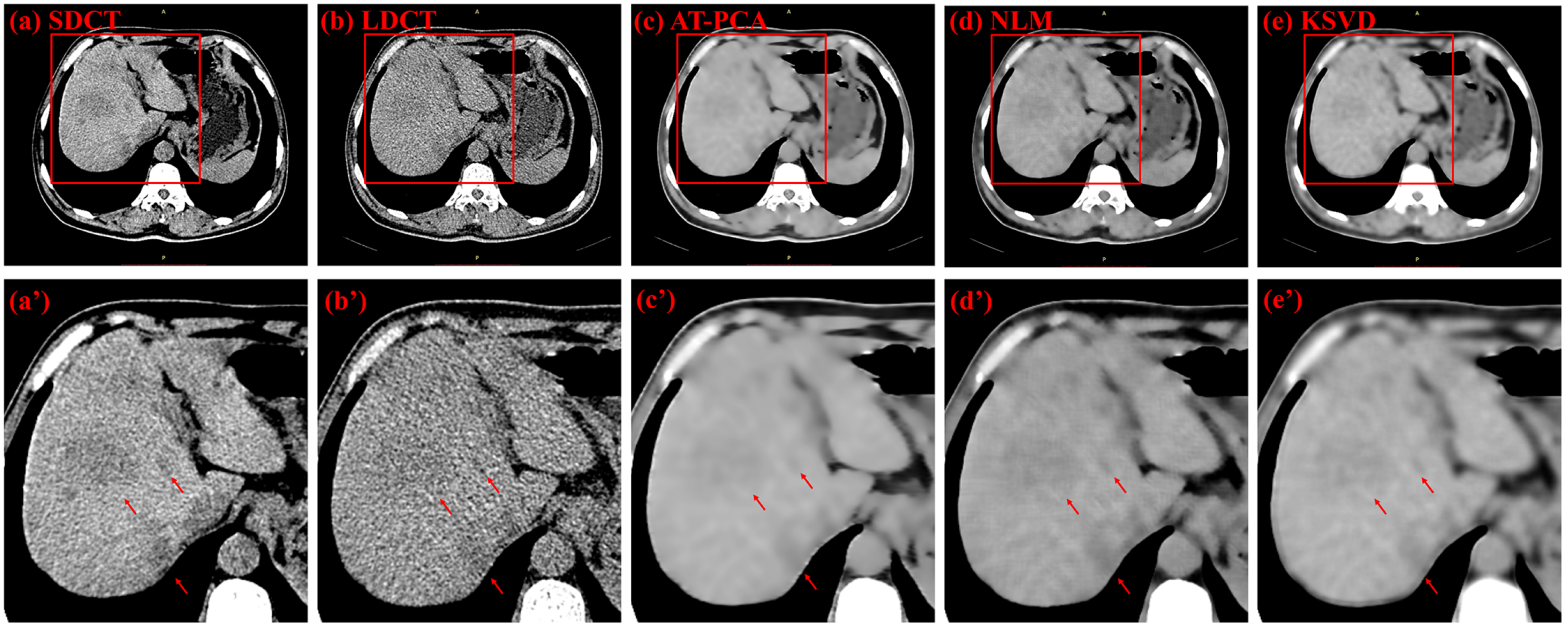
Denoising results for a 56-year-old male patient with multiple hepatic metastases (slice 2). The first column from (a) to (e) shows the SDCT image, LDCT image, and images denoised with AT-PCA, NLM, and KSVD, respectively. The second column from (a’) to (e’) shows the zoomed regions corresponding to (a) to (e).

Five different unprocessed and processed CT images scanned from a 56-year-old male patient with multiple hepatic metastases. Two discrete slices of the same person are displayed in Figs 9 and 10. The SDCT and LDCT images are shown in Figs 9a,b and 10a,b. Our proposed method [Figs 9c and 10c] is compared with popular denoising methods, such as NLM [Figs 9d and 10d] and K-SVD [Figs 9e and 10e]. NLM utilizes the similarity among several patches and reduces the redundancy of these patches [28], whereas K-SVD finds the best dictionary to represent the image as sparse compositions [29]. These two methods have achieved efficient denoising results in many image processing fields. The second columns of Figs 9 and 10 are the zoomed regions corresponding to (a) to (e). Hepatic metastases can be clearly shown in the AT-PCA based method; the noise is suppressed, and the edge of the organ is enhanced. NLM can indeed reduce the noise, but some of the new artifacts are introduced as the zoomed region [Figs 9d’ and 10d’]. The reason may be that weights decided by distance are not suitable for the similarity estimation of pixels in the image space because not all the pixels near the pixel being filtered are similar to the target pixel. For KSVD, the effect of denoising is not as good as that of AT-PCA. Meanwhile, the edges are blurred and artifacts are drawn closer to the edge of organs [Figs 9e’ and 10e’]. The reason may be that only one dictionary is utilized to remove the noise of the whole image, which is not adaptive to the specific detail information.

**Fig 11:**
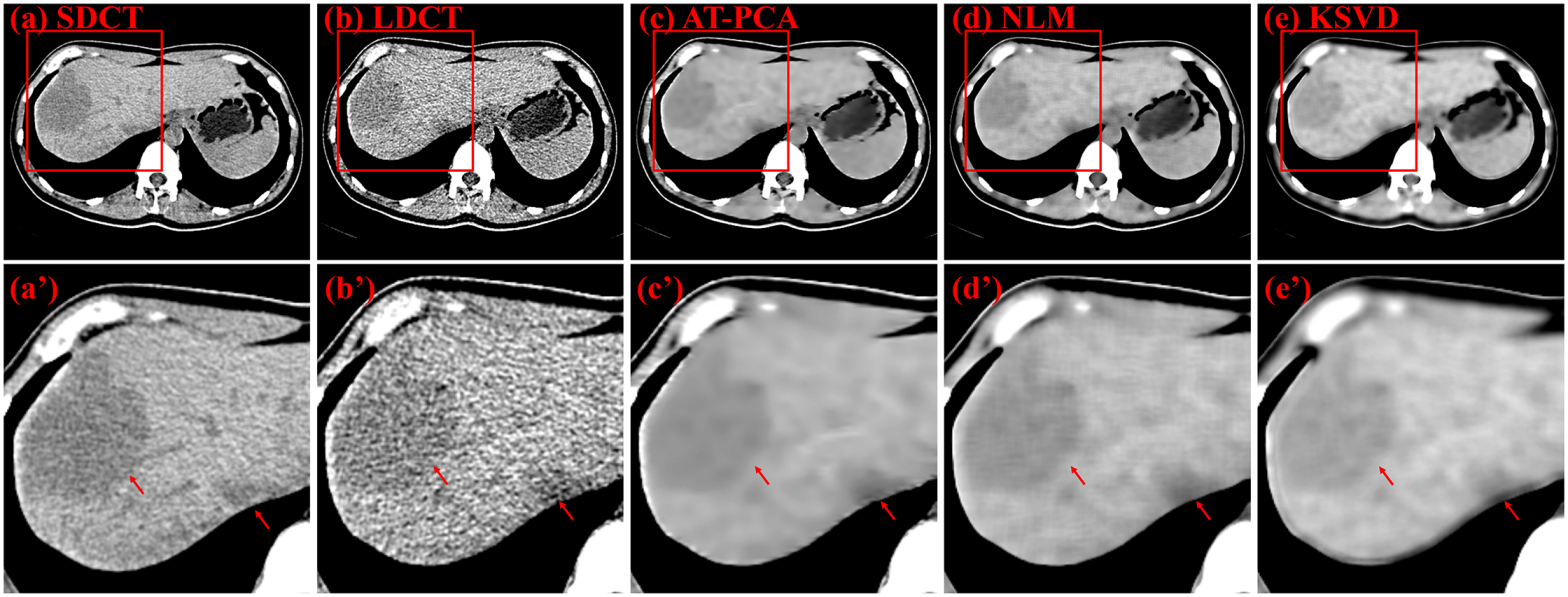
Denoising results for a 61-year-old female patient with liver tumors. The first column from (a) to (e) shows the SDCT image, LDCT image, and images denoised with AT-PCA, NLM, and KSVD, respectively. The second column from (a’) to (e’) shows the zoomed regions corresponding to (a) to (e).

Fig 11 shows the denoising results for a 61-year-old female patient with liver tumor. This is a different slice from that in Fig 7. The first column from (a) to (e) shows the SDCT image, LDCT image, and images denoised with AT-PCA, NLM and KSVD, respectively. The second column from (a’) to (e’) shows the zoomed regions corresponding to (a) to (e). Particularly, in the case of liver tumor in Fig 11, the proposed AT-PCA processed image allows a better discrimination of lesions (arrows in figures) compared with the original LDCT image and the NLM and KSVD processed image.

## Conclusions and Discussion

This paper described and applied a novel image denoising framework to suppress the pixel noise of the low-dose CT image. The algorithm of adaptive tensor-based principal component analysis is proposed in which corresponding parameters can be automatically decided for each specific noise-disturbing image. Local image structure is retained by presenting each pixel with its nearby neighbors, which are modeled as a patch. Denoising of this pixel estimates the variable of its noisy observations within similar patches. An adaptive searching window is calculated in which the similar patches are selected and very diverse structures are excluded. Tensor-based PCA are directly used on each similar patch group to adaptively train the transformation matrices on each patch mode. Clean coefficients from local principal components are adaptively shrunk by using the linear minimum mean square error method and then employed for patch reconstruction to obtain noise removal patches. After all the reconstructed patches are aggregated, the denoised image is finally obtained. The refinement is made by iterating the denoising procedure. Two types of experiments are implemented for investigation. For standard test images, the proposed AT-PCA effectively removes the noise and enhances the edge. Double denoising procedures are necessary for refinement. For clinical images, AT-PCA is compared with NLM and KSVD methods. AT-PCA showed significantly improved denoising effect. The noise is obviously removed, edges are strengthened, and no extra artifact is introduced.

The current method sequentially reduces the noise in each pixel presented by a patch. The whole denoised image is obtained after all pixels are denoised. To accelerate the denoising process, a parallel processing architecture for the proposed method should be achieved. Thus, a graphical processing unit (GPU) with parallel processing architecture will be used in our future work. We will formulate the adaptive denoising process on the GPU to increase computer speed.
